# Microbial beginnings: determinants and disruptions of the neonatal microbiome

**DOI:** 10.3389/fmicb.2025.1672780

**Published:** 2025-10-10

**Authors:** Jhommara Bautista, Andrés López-Cortés

**Affiliations:** Cancer Research Group (CRG), Faculty of Medicine, Universidad de Las Américas, Quito, Ecuador

**Keywords:** perinatal microbiome, immune development, vertical transmission, dysbiosis, precision medicine

## Abstract

The perinatal period is a critical window in human development, during which the neonatal microbiome, shaped by maternal, environmental, and clinical factors, influences immune, metabolic, and neurodevelopmental processes. Early-life microbial assembly is an active, multisite, and functionally significant phenomenon, modulated by delivery mode, feeding practices, maternal microbiota, and antibiotic exposure. Vertical microbial transmission from the maternal gut, vagina, skin, and breast milk contributes to the colonization of the infant with taxa such as *Bifidobacterium* and *Lactobacillus*, while disruptions associated with cesarean section, formula feeding, or antibiotic use have been linked to persistent dysbiosis, impaired immune maturation, and increased risk of inflammatory, metabolic, and neurodevelopmental conditions. Recent studies also challenge the sterile womb paradigm, suggesting that prenatal microbial signals, whether microbes or metabolites, may reach the maternal–fetal interface and affect fetal programming. Furthermore, neonatal microbial profiles have been associated with later-life health trajectories, suggesting exploratory value as research biomarkers; however, these associations remain preliminary and are not validated for clinical application. In this review, we summarize and integrate evidence from multiomic, clinical, and experimental studies to describe the determinants, developmental dynamics, and health consequences of the neonatal microbiome. We also highlight emerging microbiome-targeted approaches, including maternal and neonatal probiotics, nutritional modulation, and systems biology frameworks, that may help to optimize early development and reduce disease risk. Understanding and modulating the perinatal microbiome represents a promising avenue for precision medicine and early-life prevention strategies.

## Introduction

The perinatal period represents a critical window in human development during which the foundations of long-term health are established through complex host–microbe interactions. It is increasingly evident that the early-life microbiome is not a passive byproduct of environmental exposure but rather a dynamic and biologically active system that influences immune priming, metabolic programming, and neurodevelopmental maturation. The neonatal gut microbiome, in particular, undergoes rapid succession in the first weeks and months of life, shaped by a wide array of maternal and environmental factors that can have lasting consequences across the lifespan (Bautista et al., 2025c; Kalbermatter et al., 2021; Suárez-Martínez et al., 2023; Selma-Royo et al., 2024).

Among the most influential determinants of neonatal microbial assembly are mode of delivery, gestational age, infant feeding practices, antibiotic exposure, and maternal microbiome composition. Vaginal delivery facilitates vertical microbial transmission from the maternal vaginal and intestinal tracts, seeding the neonate with beneficial taxa such as *Lactobacillus*, *Bacteroides*, and *Bifidobacterium*. In contrast, cesarean section leads to initial colonization by skin-associated and hospital-derived microbes such as *Staphylococcus* and *Streptococcus*, resulting in reduced microbial diversity and delayed maturation (Tian L. et al., 2023; Zhu Y. et al., 2024). These microbial disparities are not merely compositional; they alter microbial function, immune activation, and disease susceptibility. For instance, C-section infants exhibit reduced colonization by immunoregulatory bacteria and increased risk for asthma, obesity, and type 1 diabetes later in life (Pärnänen et al., 2018; Thänert et al., 2022; Gao and Wang, 2023).

Feeding mode also plays a pivotal role in microbial development. Human milk provides more than nutrition; it delivers immunoglobulins, prebiotic oligosaccharides, and live microbes that promote colonization by *Bifidobacterium* and *Lactobacillus*, taxa associated with barrier integrity and immune homeostasis (Fouhy et al., 2019; Catassi et al., 2024; Leech et al., 2024). Formula-fed infants, in contrast, often exhibit higher microbial diversity early in life, with enrichment of facultative anaerobes and opportunistic pathogens. While the addition of synthetic human milk oligosaccharides (HMOs) and probiotics to formula has improved its bifidogenic potential, significant differences remain in the structure and function of the gut microbiome compared to breastfed infants (Dunn et al., 2017; Rasmussen et al., 2020; Coelho et al., 2021).

Vertical transmission from maternal body sites, including the gut, vagina, skin, and breast milk, contributes directly to infant microbial colonization. Approximately 50–60% of the infant gut microbiome is maternally derived, particularly from the maternal gut microbiota, which serves as the dominant reservoir for persistent neonatal strains (Wampach et al., 2018; Reyman et al., 2019; Lai et al., 2024). Recent metagenomic studies suggest the existence of a gut-mammary axis through which maternal gut microbes translocate to breast milk and reach the infant gut, reinforcing microbial continuity and function (Stewart et al., 2017; Li et al., 2018). The vaginal microbiota also plays a central role, with community state types in the mother predicting neonatal colonization patterns and influencing immune development (Thänert et al., 2022; Zhu B. et al., 2024).

Compelling evidence now suggests that microbial imprinting may begin even before birth. Although the sterile womb paradigm has historically dominated, recent studies report microbial DNA in placental tissue, amniotic fluid, and meconium, pointing to potential in utero exposures that may influence fetal immune and metabolic programming (Ho et al., 2018; Borewicz et al., 2019; Davis et al., 2022). These signals, whether live microbes or microbial metabolites, likely traverse the maternal-fetal interface and shape early immune tolerance and organogenesis. Maternal stress, diet, metabolic status, and antibiotic use during pregnancy have all been implicated in altering the in utero environment and, consequently, the early-life microbiome (Fouhy et al., 2019; Suárez-Martínez et al., 2023; Tian M. et al., 2023).

The consequences of disrupted microbial colonization during this critical period are increasingly being recognized. Early-life perturbations, including antibiotic exposure, cesarean section, or formula feeding, have been associated with heightened risks of inflammatory diseases, metabolic syndromes, neurodevelopmental delays, and even adverse aging trajectories (Fouhy et al., 2019; Thänert et al., 2022; Selma-Royo et al., 2024). Microbial biomarkers present in neonatal stool or meconium are now being explored as predictors of future disease risk, and therapeutic strategies aimed at restoring or enhancing microbial development, such as probiotics, maternal microbiota optimization, and postbiotic supplementation, are gaining traction (Pärnänen et al., 2018; Catassi et al., 2024; Zhu B. et al., 2024).

Importantly, perinatal microbiome development is strongly context-dependent, shaped by geographic and socioeconomic conditions. Cohorts from low- and middle-income countries (LMICs), such as those within the MAL-ED consortium, demonstrate that high rates of enteropathogen exposure, environmental enteric dysfunction, and undernutrition alter microbial assembly and blunt vaccine responses (Tickell et al., 2019; Cowardin et al., 2023). Clinical practices also vary widely across regions: cesarean section prevalence ranges from <15 to >50%, NICU antibiotic prescribing is often more prolonged and empiric in resource-limited settings, and breastfeeding initiation and exclusivity rates are strongly influenced by cultural and policy contexts (Prusakov et al., 2021). Beyond clinical factors, sanitation, water quality, and crowding shape early microbe–host interactions and may entrench dysbiosis in ways rarely captured in high-income settings. Yet, global microbiome research remains disproportionately focused on high-income populations, limiting the generalizability of biomarkers and interventions. Expanding representation from LMICs and tailoring microbiome-targeted strategies to diverse health systems are therefore essential for equity-oriented discovery and implementation (Blake, 2024).

**Figure 1:**
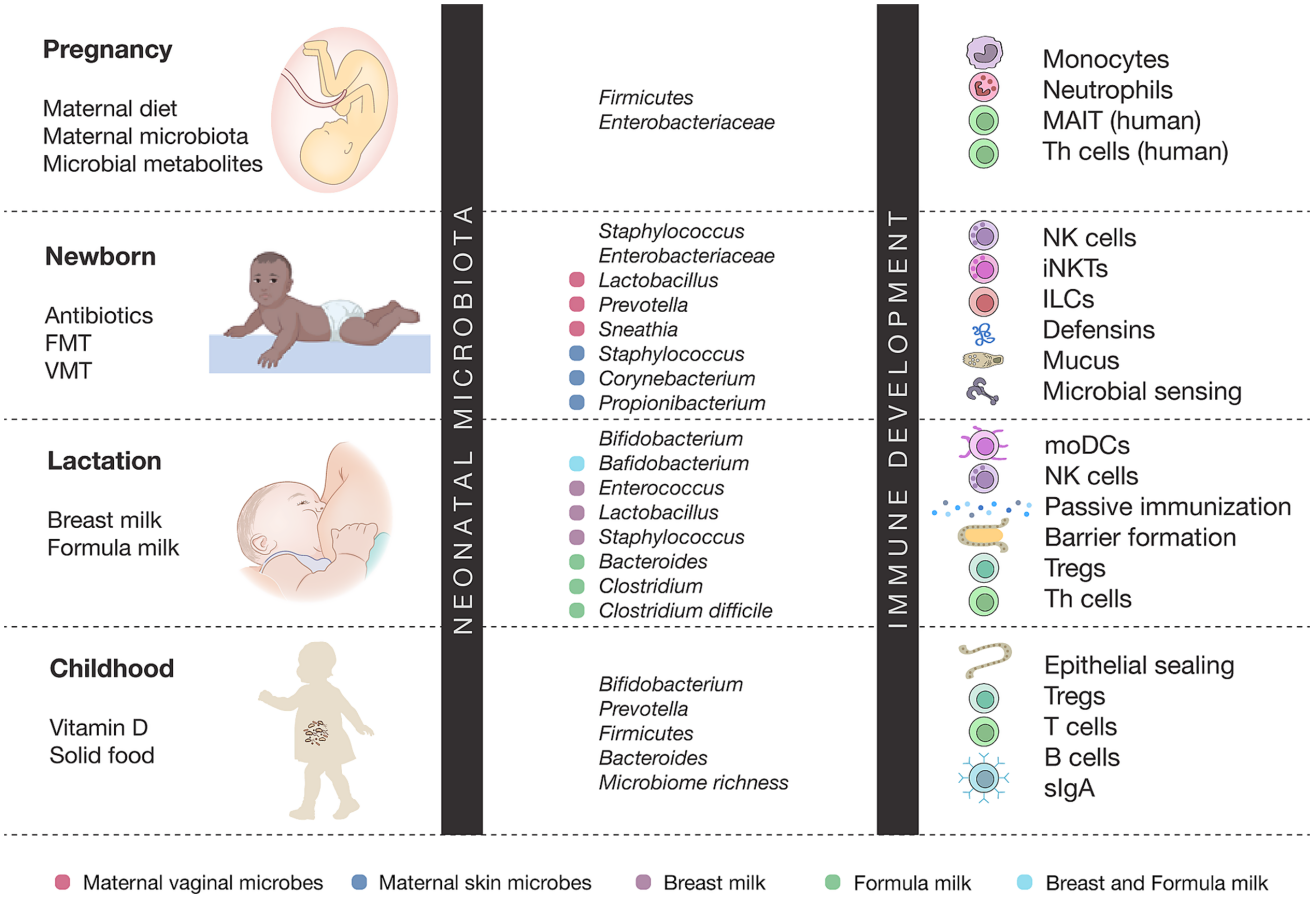
Microbiota–immune interactions across early life development. This figure depicts the dynamic interplay between microbial colonization and immune maturation from pregnancy through childhood. Maternal factors, early-life exposures, and diet shape stage-specific microbiota, which in turn influence the development of immune cells such as monocytes, NK cells, Tregs, Th cells, and mucosal defenses. Puberty is characterized by increased microbial diversity and immune imprinting, whereas adulthood is marked by disease-specific microbiota–immune associations.

In this review, we summarize and integrate emerging knowledge on the maternal and environmental determinants of the neonatal microbiome, the long-term implications of early microbial assembly, and the therapeutic potential of perinatal interventions. By integrating insights from multiomic studies, clinical trials, and mechanistic models, we highlight how targeting the perinatal microbiome offers a promising avenue to reprogram developmental trajectories and reduce the global burden of noncommunicable diseases (see Figure 1).

## Pregnancy: prenatal microbial imprinting and maternal determinants

The origin and development of the neonatal microbiome are increasingly understood as processes that begin before birth, influenced by a complex interplay of maternal, environmental, and intrauterine factors. Traditionally, the “sterile womb” hypothesis held that microbial colonization commenced only at delivery. However, this notion has been challenged by recent metagenomic evidence revealing microbial DNA and structures in the placenta, amniotic fluid, umbilical cord blood, and meconium, suggesting that initial microbial exposures may occur in utero (Collado et al., 2016; Senn et al., 2020).

Several studies have identified bacterial signatures in fetal compartments such as the placenta and amniotic fluid, most commonly from the phylum *Proteobacteria, Firmicutes*, and *Actinobacteria*, indicating potential vertical transmission pathways (Collado et al., 2016; Park et al., 2023). Although these microbial communities are of low biomass and their viability remains debated, their consistent detection across studies implies that maternal microbes may reach the fetal gut before birth, possibly through translocation from the maternal gut via immune cells or bloodstream (Senn et al., 2020; Puglisi et al., 2025).

Maternal physiological changes during pregnancy, including hormonal, metabolic, and immunological, profoundly remodel the gut and vaginal microbiota. For instance, shifts in the maternal gut microbiota across trimesters include a decline in *Firmicutes* and a relative increase in *Bacteroidetes* and *Proteobacteria*, coupled with functional enrichment in carbohydrate and lipid metabolism pathways (Puglisi et al., 2025). These dynamic changes may influence the in utero environment through microbial metabolites such as SCFAs, which are known to cross the placenta and modulate fetal immune and metabolic development (Puglisi et al., 2025; Walker, 2017).

Gestational age also critically shapes the timing and nature of microbial colonization. Preterm infants often show delayed and altered colonization patterns with a predominance of potentially pathogenic bacteria such as *Staphylococcus, Klebsiella,* and *Enterococcus*, in part due to the immaturity of the gut, frequent antibiotic use, and reduced exposure to maternal microbes (Senn et al., 2020; Farinella et al., 2022). Meconium studies support these findings, showing that prenatal factors such as gestational age and maternal health status can influence its microbial composition. Nonetheless, recent evidence suggests that perinatal factors, especially delivery mode and antibiotic exposure, exert a stronger influence than prenatal factors on meconium microbiota (Turunen et al., 2024).

Maternal stress, nutrition, and immune status during gestation have also emerged as significant modulators of the neonatal microbiome. Chronic prenatal stress can alter maternal immune regulation and gut microbiota, thereby affecting microbial and inflammatory profiles at the maternal-fetal interface (Antonson et al., 2020). These changes may predispose the offspring to neurodevelopmental and metabolic disorders, highlighting the importance of maternal homeostasis during pregnancy. Additionally, maternal dietary patterns, especially those rich in fiber or high in fat, can shape microbial populations in the maternal gut and placenta, with downstream effects on fetal microbial exposure (Puglisi et al., 2025; Zhu B. et al., 2024). Emerging evidence further suggests that microbiome development exhibits sex-specific trajectories from the perinatal period. Male and female infants differ in microbial composition and metabolite profiles, particularly in bile acid metabolism and immune-related taxa, which may contribute to sex-biased risks for autoimmunity, allergy, and neurodevelopmental disorders (Markle et al., 2013; Org et al., 2016; Laue et al., 2022). Prenatal exposures such as maternal obesity or stress appear to interact with fetal sex to differentially influence microbial seeding, underscoring the need to incorporate sex as a biological variable in microbiome research.

Although the precise mechanisms of microbial transmission to the fetus remain incompletely understood, the accumulating data support a model where prenatal microbial signals, whether live microbes, microbial DNA, or metabolites, can influence early gut colonization and immune education. Such prenatal programming of the microbiome may have long-lasting consequences on disease susceptibility, including risks for allergy, asthma, obesity, and neurodevelopmental disorders (Walker, 2017; Kim et al., 2024). Recent insights highlight how gut microbiota dynamics and immune signaling via the gut-brain-immune axis, suggesting that prenatal microbial programming and subsequent dysbiosis may modulate neuropathological outcomes (Bautista et al., 2025b). In summary, prenatal factors, including maternal microbiota dynamics, gestational age, stress, immune function, and nutritional status, contribute to the early microbial imprinting of the fetus. While direct microbial colonization in utero remains debated, it is increasingly evident that prenatal exposures shape the trajectory of neonatal microbiome development and health outcomes.

### Newborn: birth mode, vertical transfer, and immediate immune priming

The mode of birth plays a pivotal role in shaping the initial microbial landscape of the neonate, influencing not only microbial composition but also functional development and immune priming. Vaginal delivery exposes the newborn to maternal vaginal, intestinal, and perineal microbiota, facilitating the vertical transfer of commensal bacteria such as *Lactobacillus*, *Bacteroides*, and *Bifidobacterium*, which are foundational for a healthy gut ecosystem and immune development (Rasmussen et al., 2020; Coelho et al., 2021; Leech et al., 2024). In contrast, cesarean section (C-section) bypasses the birth canal, resulting in initial colonization by skin-associated and hospital-derived taxa, including *Staphylococcus*, *Streptococcus*, and *Clostridium* species, often leading to reduced microbial diversity and delayed microbial maturation (Dunn et al., 2017; Coelho et al., 2021; Lai et al., 2024).

Metagenomic and immune stimulation studies have revealed that these early microbial differences are not merely compositional. Infants born vaginally acquire maternal-specific microbial strains that confer distinct functional capacities, including enhanced biosynthesis of immunostimulatory molecules such as lipopolysaccharides (LPS). These microbial products induce higher levels of pro-inflammatory cytokines such as TNF-*α* and IL-18, promoting early immune system priming (Wampach et al., 2018). The absence of such maternal strain transfer in C-section neonates may result in lower levels of these cytokines and a reduced capacity to stimulate innate immune responses during the critical early window of immune development (Lai et al., 2024; Wampach et al., 2018).

Epidemiological data support these mechanistic insights. Cesarean-born infants exhibit an increased risk for immune-mediated conditions, including asthma, allergies, and type 1 diabetes, which may be rooted in the altered trajectory of microbial colonization and immune programming (Rasmussen et al., 2020; Coelho et al., 2021; Leech et al., 2024; Reyman et al., 2019). Even when accounting for intrapartum antibiotic exposure, delivery mode independently shapes microbiome composition. At 6 weeks postpartum, cesarean-born infants demonstrate significantly lower abundances of *Bacteroides* and *Bifidobacterium*, both taxa associated with immunoregulatory functions and metabolic health, while exhibiting shifts in microbial metabolic pathways involved in short-chain fatty acid (SCFA) production and mucosal integrity (Leech et al., 2024).

The influence of delivery mode on microbiome composition is also evident across other body sites. Oral microbiota studies show significant differences in colonization between birth modes, with vaginally delivered neonates harboring a greater abundance of *Prevotella*, *Lactobacillus*, and *Gardnerella*, whereas cesarean-born infants show higher levels of environmental and skin-associated genera like *Pseudomonas* and *Staphylococcus* (Li et al., 2018). Importantly, in preterm infants, some studies indicate that long-term microbiome trajectories may converge irrespective of birth mode, largely due to the dominant influence of Neonatal Intensive Care Unit (NICU) exposures and widespread antibiotic administration (Stewart et al., 2017), in healthy term infants, the effects of birth mode persist for months and can influence clinical outcomes such as susceptibility to respiratory infections (Rasmussen et al., 2020).

The vertical transmission of microbes from mother to offspring is a fundamental process that shapes the establishment and developmental trajectory of the neonatal microbiome. This transfer occurs through multiple maternal body sites, including the vagina, gut, skin, oral cavity, and breast milk, and influences microbial colonization across diverse infant niches such as the gut, oral cavity, respiratory tract, and skin (Bogaert et al., 2023; Ferretti et al., 2018; Tian M. et al., 2023).

Strain-resolved metagenomic studies have revealed that approximately 58% of the infant microbiome can be attributed to maternal sources, with the maternal gut microbiome serving as the dominant contributor of persistent microbial strains to the infant gut (Bogaert et al., 2023; Ferretti et al., 2018). Specifically, maternal gut-derived strains such as *Bacteroides*, *Bifidobacterium*, and *Escherichia-Shigella* are commonly detected in both breast milk and the infant gut, suggesting that maternal fecal microbes may translocate to the mammary gland through immune-mediated pathways, a process referred to as the gut-mammary axis (Giugliano et al., 2025; Meng et al., 2025; Zeng et al., 2025).

Breast milk serves as a critical vector for maternal microbial transfer. Recent studies show that 25–30% of the bacterial taxa in the infant gut are traceable to maternal breast milk, and up to 22% of the breast milk microbiota itself originates from the maternal gut (Meng et al., 2025). This bidirectional relationship is not merely compositional; breast milk microbes exert functional effects by promoting immune system maturation and protecting against pathogenic colonization during early life. Vertical transmission via milk includes beneficial genera such as *Lactobacillus*, *Bifidobacterium*, and *Streptococcus*, which influence metabolic and immunological development in the infant (Meng et al., 2025; Jašarević et al., 2021; Gomes et al., 2024).

The vaginal microbiota also plays a central role in maternal–infant microbial transmission, particularly during vaginal delivery. Dominant community state types (CSTs) in the maternal vagina, especially those rich in *Lactobacillus crispatus*, *Gardnerella vaginalis*, and *Atopobium vaginae*, are among the first colonizers of the neonatal gut and mucosal surfaces (Jašarević et al., 2021; Bhattacharyya et al., 2023). Animal models have confirmed that vaginal microbial exposure at birth produces long-term effects on immune programming, brain development, and metabolic function, and that these effects are modulated by the maternal intrauterine environment, such as diet-induced obesity or vaginal dysbiosis (Jašarević et al., 2021).

Importantly, vertical microbial inheritance extends beyond the bacteriome. Multi-kingdom metagenomic studies reveal that bacteriophages and eukaryotic viruses (virome), fungi such as *Candida* and *Malassezia* (mycobiome), and archaea like *Methanobrevibacter* also co-colonize the neonate and contribute to immune imprinting, pathogen defense, and metabolic programming (Ferretti et al., 2018; Zeng et al., 2025). Phages regulate bacterial succession and resistome dynamics through predation and lysogeny, fungal ligands engage Dectin-1 and TLR pathways to calibrate neutrophil and Th17 responses, and methanogens reshape redox balance and cross-feeding networks that influence SCFA and bile-acid signaling. These cross-domain interactions highlight that maternal microbial transfer is a coordinated, multi-kingdom process with durable effects on neonatal health trajectories.

While the maternal gut and vaginal microbiomes are primary sources, other body sites such as the oral cavity and skin also contribute transiently to infant colonization, particularly during skin-to-skin contact and breastfeeding (Ferretti et al., 2018; Russell et al., 2023). These transient colonizers may facilitate niche-specific seeding of the oral and dermal microbiota, contributing to microbial diversity across body compartments.

In summary, the maternal microbiome is not a passive background feature of pregnancy and lactation but an active, dynamic source of microbial and functional inheritance. Recognizing the contribution of the virome, mycobiome, and archaea enriches this framework, underscoring that neonatal colonization is shaped by multi-kingdom ecosystems rather than bacteria alone. Understanding the mechanisms and timing of maternal microbial transfer opens new opportunities for microbiome-targeted interventions aimed at optimizing neonatal development and reducing disease risk later in life.

### Lactation and microbial developmental pathways

The type of early-life feeding exerts a profound influence on the assembly and functional maturation of the infant gut microbiota, with implications for growth, immune development, and long-term health. Breastfeeding is widely recognized as the optimal mode of infant feeding, promoting the establishment of a gut ecosystem enriched in *Bifidobacterium* and *Lactobacillus*, which are associated with protective, anti-inflammatory, and immunomodulatory properties (Borewicz et al., 2019; Davis et al., 2022; Odiase et al., 2023). In contrast, formula feeding is often associated with increased microbial diversity at early time points, greater abundance of adult-like taxa such as *Clostridium*, *Enterobacteriaceae*, and *Veillonella*, and altered microbial metabolic pathways (Odiase et al., 2023; Ma et al., 2020; Ho et al., 2018).

Human milk is not merely a source of nutrients but a complex bioactive matrix that includes immunoglobulins, antimicrobial peptides, HMOs, and a rich array of maternal-derived bacteria (Borewicz et al., 2019; Davis et al., 2022; Boudry et al., 2021). HMOs, in particular, serve as selective substrates for *Bifidobacterium* species, shaping a microbiota that supports mucosal integrity and modulates immune responses. These oligosaccharides are absent in conventional formulas, which limits their bifidogenic and immunological capacities (Boudry et al., 2021; Bakshi et al., 2023).

Studies have consistently demonstrated that exclusively breastfed infants exhibit lower *α*-diversity but a microbiome more specialized for fermenting HMOs, promoting colonization by beneficial anaerobes and suppressing the expansion of opportunistic pathogens (Odiase et al., 2023; Ma et al., 2020; Ho et al., 2018). The gut of formula-fed infants, especially those consuming traditional or non-fortified formulas, tends to harbor higher abundances of *Escherichia coli*, *Clostridium difficile*, and *Enterococcus*, taxa associated with increased risk for inflammatory and metabolic disorders (Odiase et al., 2023; Ma et al., 2020; Bakshi et al., 2023). Recent advances in formula design have attempted to mimic the composition of human milk by incorporating prebiotics, such as galacto-oligosaccharides (GOS) and fructo-oligosaccharides (FOS), and even synthetic HMOs (Bakshi et al., 2023; Borewicz et al., 2019). These modifications have shown bifidogenic effects and can partially restore microbiota profiles resembling those of breastfed infants. However, significant differences in the dynamics and functional trajectories of microbial development remain, particularly in the early weeks of life (Borewicz et al., 2019).

Breastfeeding also supports vertical microbial transfer, with viable strains of *Bifidobacterium breve* and *Lactobacillus plantarum* being simultaneously isolated from breast milk and infant feces, highlighting a direct maternal influence on neonatal microbial seeding (Murphy et al., 2017). Moreover, maternal diet, lactation stage, and milk handling practices further modulate the milk microbiome and, consequently, infant gut colonization (Boudry et al., 2021; Lok et al., 2025).

From a clinical perspective, exclusive breastfeeding has been associated with lower incidence of diarrhea, respiratory infections, and allergic diseases, and has shown protective effects against obesity and type 2 diabetes later in life, likely mediated through microbiome-immunological pathways (Ho et al., 2018; Davis et al., 2022; Li et al., 2025). In contrast, non-breastfed infants show increased susceptibility to infections and altered metabolic programming. Breastfeeding fosters a microbial developmental pathway characterized by selective enrichment of beneficial taxa, lower microbial diversity, and functional specialization aligned with immune maturation and barrier protection. Formula feeding, despite recent innovations, induces a divergent microbial trajectory with potential long-term consequences. These findings reinforce the critical importance of early nutritional choices in shaping microbial ecology and health outcomes during the foundational period of life.

Beyond compositional differences, early nutritional choices also translate into distinct immune programming. HMOs fermented by *Bifidobacterium* generate SCFAs that expand Foxp3^+^ Tregs via GPR43/GPR109A signaling and histone deacetylase (HDAC) inhibition, enhancing IL-10, TGF-*β*, and IgA class switching (Furusawa et al., 2013; Koh et al., 2016). Breast milk oligosaccharides and tryptophan-derived indoles calibrate Th17 and ILC3 activity through AhR signaling, sustaining IL-22–mediated barrier repair (Yang et al., 2022). Riboflavin-derived MR1 ligands expand mucosal associated invariant T (MAIT) cells, while tonic LPS and cytokine cues (IL-12/IL-15) educate NK function (Hinks and Zhang, 2020). Collectively, these lineage-specific pathways illustrate how breastfeeding strengthens tolerance, barrier immunity, and antiviral tone, whereas formula feeding or early disruption may blunt these immune programs and predispose to allergy, infection, and metabolic disease later in life (Rosas-Salazar et al., 2022).

### Childhood: ecological maturation, functional trajectories, and health signals

The neonatal period represents a critical window during which microbial colonization patterns may set the foundation for long-term health or predisposition to disease. Mounting evidence from longitudinal, epidemiological, and mechanistic studies indicates that specific microbial profiles during early life correlate with immediate developmental milestones and may serve as investigational indicators of metabolic, immunological, and neurodevelopmental trajectories; prospective validation and clinical calibration are still needed (Derrien et al., 2019; Neu and Stewart, 2025).

During infancy, the gut microbiota undergoes rapid succession, typically transitioning from dominance by facultative anaerobes such as *Enterobacteriaceae* and *Streptococcus* to obligate anaerobes including *Bifidobacterium* and *Faecalibacterium*. This ecological maturation process is critical for immune system education, nutrient absorption, and resistance to pathogens (Lv et al., 2022; Wang et al., 2024). Deviations from this trajectory, such as delayed colonization by *Bifidobacterium*, reduced microbial diversity, or sustained dominance by pro-inflammatory taxa, have been linked to increased risks of allergic diseases, obesity, type 1 diabetes, and neurodevelopmental disorders (Lv et al., 2022; Neu and Stewart, 2025; Wilmanski et al., 2021).

Neonatal microbial profiles, particularly those established in the first weeks of life, can function as biomarkers for later health outcomes. For instance, specific taxa found in meconium have been associated with cognitive and social behavioral development at six months of age, as shown in the Rio Birth Cohort study. Infants exhibiting lower microbial alpha diversity and enrichment in *Ruminococcaceae*, *Treponema*, and *Eubacterium* in meconium had higher risks of delayed social development (Naspolini et al., 2025). These findings support the concept that early microbial imbalances may influence brain development through mechanisms involving microbial metabolites, immune signaling, and the gut-brain axis. Recent studies expand this view, showing that neonatal microbiota regulate the gut–brain–immune axis through multiple pathways. SCFAs and indole derivatives promote blood–brain barrier integrity and microglial maturation, while vagal signaling and cytokine networks mediate bidirectional communication between the gut and developing brain (Wang et al., 2023; O’Riordan et al., 2025). Cohort analyses demonstrate that microbiome “maturity” in infancy correlates with improved cognitive outcomes and behavioral development, whereas dysbiosis is associated with higher risks for autism spectrum disorders and anxiety-related traits (Bautista et al., 2025b; Ronan et al., 2021; Lu and Claud, 2019). These findings highlight the neonatal microbiome as a key regulator of neurodevelopmental trajectories via immune–neural crosstalk.

Functional alterations in neonatal microbial communities also carry predictive value. Multi-omics analyses have revealed that the presence of genes related to vitamin synthesis, amino acid metabolism, and anti-inflammatory pathways during early microbial colonization are associated with enhanced metabolic resilience and immune regulation later in life (Neu and Stewart, 2025; Wang et al., 2024). Conversely, disruptions in these functional profiles, often due to cesarean section birth, antibiotic exposure, or formula feeding, can perturb immune homeostasis and promote chronic inflammation (Lv et al., 2022; Hickman et al., 2024). Beyond SCFAs, emerging evidence highlights the importance of other microbial metabolites in shaping neonatal physiology. Tryptophan-derived indoles such as indole-3-lactic acid activate the aryl hydrocarbon receptor (AhR), modulating Treg/Th17 balance, barrier maturation, and mucosal tolerance (Henrick et al., 2021; Laursen et al., 2021). Likewise, secondary bile acids produced by microbial metabolism signal through FXR and TGR5, influencing dendritic cell function, systemic inflammation, and metabolic programming (Godlewska et al., 2022). These pathways, increasingly detected through metabolomic analyses of neonatal stool and breast milk, suggest that microbial metabolites constitute key mediators of host–microbiome communication during early life.

Emerging data also indicate that microbial trajectories in infancy may shape aging-related outcomes. Longitudinal studies have demonstrated that individuals with healthier neonatal microbiome signatures, characterized by high microbial diversity and early colonization by beneficial commensals, are more likely to maintain diverse and metabolically active microbiota profiles into older age, which correlates with reduced frailty and increased survival (Ghosh et al., 2022; Wilmanski et al., 2022, 2021). A particularly intriguing finding is the observation that centenarians exhibit distinct microbial patterns not seen in younger adults, suggesting that early-life microbiome imprinting may influence the likelihood of achieving healthy longevity (Ghosh et al., 2022; Wilmanski et al., 2022). In summary, the neonatal microbiome is more than a transient developmental feature; it represents a dynamic and programmable system whose structure and function can forecast future health or disease risk. The identification of microbial biomarkers in early life opens promising avenues for preventive strategies, including nutritional modulation, probiotic supplementation, and personalized microbial interventions aimed at optimizing long-term resilience.

### Antibiotic exposure and microbial disruption in early life

Antibiotic administration during early life, particularly in the perinatal period encompassing pregnancy, delivery, and infancy, can profoundly alter the developmental trajectory of the gut microbiome (Schwartz et al., 2020; Huang et al., 2024). This is a critical period when microbial colonization patterns are being established and synchronized with immune, metabolic, and neurological development. Widespread antibiotic use during this window is a double-edged sword: while often necessary for treating or preventing infection, it exerts unintended ecological consequences on the infant gut microbiota. Antibiotics are among the most commonly prescribed medications to infants and young children, with estimates suggesting that 40–70% of infants in high-income countries receive at least one antibiotic course in their first year of life, and in some low and middle-income regions, children receive up to 11 courses within the first 2 years (Lebeaux et al., 2022; Thänert et al., 2022; Benitez et al., 2025).

These exposures disrupt the normal succession of microbial colonization by selectively depleting key commensals such as *Bifidobacterium* and *Lactobacillus*, and allowing for overgrowth of opportunistic bacteria like *Klebsiella*, *Enterococcus*, and *Escherichia coli*. This shift from a balanced, protective microbial ecosystem to one dominated by pathobionts is associated with a loss of alpha diversity and delayed microbial maturation (Thänert et al., 2022; Langdon et al., 2016). The degree of disruption depends on multiple factors, including the spectrum of antibiotics used, duration of treatment, gestational age at exposure, and co-existing host or environmental variables. For example, neonates treated with amoxicillin plus cefotaxime in the first week of life exhibit the most pronounced shifts in microbial composition and resistome expansion, while those treated with penicillin and gentamicin show comparatively less disruption (Reyman et al., 2022).

One of the most concerning consequences of early antibiotic exposure is the enrichment of the gut resistome, the pool of antibiotic resistance genes (ARGs) harbored by both pathogenic and commensal bacteria. These genes can be transmitted between microbial species via horizontal gene transfer, enabling the emergence of multidrug-resistant organisms. Systematic reviews and experimental models have consistently shown that antibiotic-treated infants exhibit a higher abundance and diversity of ARGs, and that these changes may persist for months to years, long after treatment cessation (Schwartz et al., 2020; Lebeaux et al., 2022; Thänert et al., 2022). This expansion of the resistome poses not only individual risks, such as increased susceptibility to future antibiotic-resistant infections, but also public health threats through the propagation of resistance genes in microbial communities.

The health implications of these microbial disruptions are far-reaching. Longitudinal studies link early-life antibiotic exposure to increased risk of obesity, asthma, allergic disorders, inflammatory bowel disease, and even neurodevelopmental conditions. For instance, a birth cohort study demonstrated that infants exposed to antibiotics in the first year of life were more likely to develop overweight or obesity by age two and a half, an effect associated with elevated levels of *Faecalibacterium*, *Agathobacter*, and *Klebsiella*, and decreased levels of *Bifidobacterium* (Li et al., 2022). These findings align with other epidemiological and mechanistic data indicating that antibiotic-induced dysbiosis compromises immune tolerance, alters gut barrier function, and promotes metabolic and inflammatory dysregulation (Thänert et al., 2022; Huang et al., 2024; Neuman et al., 2018).

Despite some evidence of partial microbiota recovery over time, complete restoration of pre-antibiotic composition and function is rare. Even short-term antibiotic exposures can lead to prolonged alterations in microbial diversity, metabolism, and ecological networks. For example, a 14-month murine study revealed that brief exposure to ceftriaxone in early life led to persistent changes in gut microbial metabolism and molecular ecological networks, with incomplete recovery of both diversity and network stability (Schwartz et al., 2020; Hong et al., 2024). Similarly, while certain studies suggest that microbiome changes may attenuate by 12 months of age, functional disruptions, particularly within the resistome, often linger, underscoring the microbiome’s limited resilience (Reyman et al., 2022),

Given these risks, there is growing interest in strategies to mitigate the impact of antibiotics on the infant microbiome. These include judicious antibiotic stewardship, particularly the avoidance of broad-spectrum agents when not clinically indicated, and the development of adjunctive interventions such as probiotics, prebiotics, or even fecal microbiota transplantation (FMT) to restore microbial homeostasis. However, the efficacy and safety of such interventions in neonates remain under investigation, and further research is essential to establish age-appropriate, evidence-based therapies (Thänert et al., 2022; Huang et al., 2024; Langdon et al., 2016).

In summary, early-life antibiotic exposure constitutes a major modifiable determinant of gut microbial development with lasting implications for host immunity, metabolism, and disease susceptibility. These perturbations not only compromise the infant microbiome’s structure and function but also expand the resistome, increasing the risk of antimicrobial resistance. A deeper understanding of the timing, dose, and spectrum of antibiotic effects, and of strategies to restore microbial balance, will be crucial for advancing precision medicine and protecting long-term child health.

### Targeting the perinatal microbiome through maternal and neonatal therapies

Therapeutic modulation of the perinatal microbiome has emerged as a promising strategy to enhance neonatal health, reduce disease risk, and promote lifelong physiological resilience. This developmental window is characterized by rapid microbial colonization, immune priming, and metabolic programming, all of which can be shaped by targeted maternal and neonatal interventions. Among the most widely studied approaches is the administration of probiotics. In preterm neonates, who often exhibit dysbiosis marked by low diversity and high abundance of pathobionts, direct probiotic supplementation has been shown to favorably alter microbial colonization (Beck et al., 2022; Tian M. et al., 2023). A randomized controlled trial demonstrated that early administration of *Lactobacillus rhamnosus GG* and *Bifidobacterium lactis* Bb-12 to neonates increased the relative abundance of beneficial taxa such as *Bifidobacterium animalis* and *Lactobacillales*, whereas maternal supplementation alone had limited effects, underscoring the superior efficacy of direct neonatal administration (Rahkola et al., 2023).

The specificity of probiotic strains is crucial in determining therapeutic outcomes. A longitudinal metagenomic analysis in very preterm infants demonstrated that different probiotic formulations, such as Infloran (*Bifidobacterium bifidum* and *Lactobacillus acidophilus*) and Labinic (*Bifidobacterium bifidum*, *Bifidobacterium longum* subsp. *infantis*, and *Lactobacillus acidophilus*), produced distinct preterm gut community types (PGCTs) (Beck et al., 2022). These PGCTs were linked to differential stool metabolite profiles and altered gene expression in host intestinal organoids, indicating that probiotic strain composition can shape both microbial ecology and host–microbe interactions in a strain-specific manner (Beck et al., 2022; Neu and Stewart, 2025). These findings support a precision medicine approach, where probiotic selection is matched to individual clinical contexts and developmental stages.

Beyond probiotics, maternal microbiome manipulation is another avenue for shaping neonatal microbial outcomes. Emerging evidence shows that spontaneous preterm birth results in a greater contribution of maternal gut microbiota to neonatal colonization compared to iatrogenic preterm birth, regardless of delivery mode. This suggests that maternal microbial composition during pregnancy significantly influences neonatal colonization patterns (Neu and Stewart, 2025; Buffet-Bataillon et al., 2021). Interventions such as maternal dietary modulation, targeted prebiotics, or probiotics may thus improve microbial transmission to the infant. Though still in experimental stages, FMT has also been proposed as a strategy to restore optimal maternal microbial communities and promote vertical transfer at birth (Buffet-Bataillon et al., 2021; Mills et al., 2025).

Mechanistically, multiomic technologies provide a powerful platform to guide these interventions. Integrating metagenomics, metabolomics, proteomics, and transcriptomics allows for a comprehensive view of the host-microbiome interface. These approaches help to clarify the causal pathways by which microbial interventions influence neonatal development and disease susceptibility. For instance, metabolomic analyses of stool samples from probiotic-treated neonates revealed shifts in functional outputs, such as SCFA production, which have direct immunomodulatory effects on T cell maturation and regulatory pathways (Neu and Stewart, 2025; Bui et al., 2025). SCFAs, particularly acetate, propionate, and butyrate, derived from microbial fermentation of HMOs, are known to reinforce gut barrier function and promote immune tolerance, particularly in the context of inflammatory conditions such as necrotizing enterocolitis (Bui et al., 2025).

The clinical translation of microbiome-targeted therapies also requires careful attention to safety, efficacy, and implementation protocols. Although probiotics are generally well tolerated, rare but serious adverse events such as probiotic-associated sepsis have been reported, particularly in immunocompromised or extremely low birth weight infants (Beck et al., 2022). Regulatory standards for microbial therapeutics in neonates remain underdeveloped, and consensus is still lacking on optimal dosing, duration, and combinations of interventions (Tian M. et al., 2023; Iqbal et al., 2023). Moreover, population-specific and context-specific variability, including variations in breastfeeding prevalence, antibiotic exposures, and NICU protocols, must be considered when designing intervention strategies (Buffet-Bataillon et al., 2021; Tian M. et al., 2023).

To facilitate clinical application, we provide a comparative summary of major microbiome-targeted strategies (Table 1). This structured overview highlights target populations, delivery routes, efficacy, strain or compound specificity, timing of administration, safety considerations, and implementation challenges, offering a translational perspective for clinicians and policymakers.

**Table 1:** Comparative overview of microbiome-targeted interventions in the perinatal period.

Intervention	Target population	Delivery route	Efficacy (evidence)	Strain/compound specificity	Timing of administration	Known risks / safety concerns	Key challenges
Probiotics (*L. rhamnosus* GG, *B. infantis*)	Preterm and term neonates; some maternal use	Oral (neonate formula, capsules); maternal oral during pregnancy/lactation	RCTs show reduced NEC in preterm infants; improved colonization with *Bifidobacterium*	Highly strain-specific; efficacy not generalizable	Neonatal administration most effective; maternal use less consistent	Rare sepsis in immunocompromised infants; mislabeling issues	Regulatory inconsistencies; variability in strain-level outcomes
Human Milk Oligosaccharides (HMOs) (synthetic or natural)	Formula-fed infants	Fortified formula	Promote *Bifidobacterium* growth; SCFA production; partial convergence with breastfed microbiota	Specific HMOs (e.g., 2′-FL, LNnT) differ in effects	Early postnatal period, ideally first 6 months	Generally safe; long-term effects still uncertain	Cost, production scalability, population variability
Maternal supplementation (diet, probiotics, prebiotics)	Pregnant and lactating women	Oral (capsules, diet)	Modest effects on vertical transfer and milk microbiota	Dependent on maternal microbiome composition	Gestation and lactation windows	Minimal adverse events reported	Inter-individual maternal variability; uncertain neonatal uptake
FMT / Vaginal seeding	C-section or high-risk neonates	Oral capsule, rectal enema, or vaginal swab	Restores maternal-like microbiota; promising but limited trials	Donor-dependent; not standardized	At birth or early neonatal period	Risk of pathogen transmission; ethical issues	No regulatory consensus; long-term outcomes unknown

NEC, necrotizing enterocolitis; HMOs, human milk oligosaccharides; SCFAs, short-chain fatty acids; 2′-FL, 2′-fucosyllactose; LNnT, lacto-N-neotetraose; FMT, fecal microbiota transplantation; NICU, neonatal intensive care unit; RCTs, randomized controlled trials.

Despite encouraging evidence, several barriers limit widespread clinical adoption. First, strain-level variability is a critical determinant of efficacy: probiotic benefits are not universal, and even within HMOs, individual compounds exert distinct effects (Beck et al., 2022; Neu and Stewart, 2025; Boudry et al., 2021; Bakshi et al., 2023). Second, regulatory oversight lags behind practice. Probiotics are often marketed as food supplements without standardized quality controls, while FMT and vaginal seeding remain under strict scrutiny (Iqbal et al., 2023; Mills et al., 2025; Zeng et al., 2025). Third, long-term tracking is scarce. Few studies follow infants beyond early childhood, leaving uncertain whether interventions confer durable immunological, metabolic, or neurodevelopmental benefits (Wilmanski et al., 2021, 2022). Finally, implementation challenges, including differences in NICU protocols, maternal diets, breastfeeding prevalence, and antibiotic use, underscore the need for context-specific strategies (Buffet-Bataillon et al., 2021; Selma-Royo et al., 2024). Addressing these issues through harmonized guidelines, long-term cohort studies, and precision intervention design will be crucial for safe and effective translation.

Future research must focus on refining microbial interventions through a systems biology lens. Multiomic data integration, longitudinal sampling, and functional validation in relevant model systems are essential to move from association to causation and identify biomarkers predictive of intervention response (Neu and Stewart, 2025; Pronovost and Hsiao, 2019). Additionally, novel therapeutic modalities, such as postbiotics, engineered commensals, and personalized synbiotics, are under development and may offer more targeted, stable, and safer alternatives to conventional probiotics (Buffet-Bataillon et al., 2021; Mills et al., 2025).

In conclusion, maternal and neonatal therapies targeting the perinatal microbiome offer a compelling opportunity to influence developmental trajectories and improve health outcomes. Direct neonatal probiotic supplementation, maternal microbiome optimization, and integrative multiomic approaches form the foundation of next-generation strategies in neonatal precision medicine. Continued research into microbial function, host-microbe communication, and therapeutic safety will be critical for advancing these interventions from bench to bedside.

## Conclusions and future perspectives

Mounting evidence underscores the critical role of the perinatal microbiome in shaping the foundations of long-term health, with profound effects on immune education, metabolic programming, and neurodevelopment (Wampach et al., 2018). The establishment of the neonatal microbiome is a dynamic and multifactorial process, primarily influenced by maternal microbial sources, birth mode, feeding practices, and environmental exposures. Disruptions during this sensitive window, especially through cesarean section, antibiotic use, or formula feeding, can result in persistent dysbiosis, increasing the risk for chronic diseases such as asthma, obesity, atopic conditions, inflammatory bowel disease, and neurodevelopmental disorders (Ma et al., 2020; Senn et al., 2020).

Maternal microbiomes, particularly from the gut, vagina, oral cavity, and skin, play a central role in seeding the neonatal gastrointestinal tract. Although traditional dogma posited that colonization begins only at birth, the detection of microbial DNA in placental tissue, amniotic fluid, and meconium has challenged the sterile womb paradigm, opening new avenues for understanding microbial priming in utero (Farinella et al., 2022; Russell et al., 2023). However, the presence of live microbes versus microbial components in these sites remains controversial, and future studies must differentiate between transient translocation events and true colonization.

Among external disruptors, antibiotic exposure in both mothers and neonates has emerged as a major disruptor of microbial assembly, often leading to the loss of key taxa and functional capacities and increasing the abundance of antibiotic resistance genes (Schwartz et al., 2020; Tian M. et al., 2023). While some resilience is observed post-exposure, recovery is variable and often incomplete, especially when perturbations occur during critical windows of development. This highlights the need for precision in antibiotic stewardship and consideration of downstream microbiome consequences in maternal and pediatric care.

Mechanistic insights reveal that microbial influences extend beyond composition to functional imprinting. Microbial metabolites such as butyrate and other SCFAs act as histone deacetylase inhibitors, enhancing histone acetylation and regulatory T cell differentiation. Folate- and one-carbon–linked pathways influence DNA methylation in neonatal immune cells, while tryptophan derivatives regulate histone modifications and transcriptional programs in developing tissues (Kopczyńska and Kowalczyk, 2024; Stein and Riber, 2023; Krautkramer et al., 2017, 2016). These findings indicate that the perinatal microbiome establishes epigenetic memories with long-term consequences.

Complementing bacterial contributions, the virome also plays a pivotal role. Oncogenic viruses such as HPV, EBV, HBV, HCV, and HTLV-1 not only drive chronic inflammation and immune evasion but also reshape the host epigenome, silencing tumor suppressors and deregulating developmental pathways (Bautista and Lopez-Cortes, 2025). These virus-induced epigenetic “scars” may persist beyond viral clearance, underscoring the importance of integrating virome research into perinatal microbiome studies. Emerging strategies that combine oncolytic virotherapy, epigenetic drugs, and immune modulation suggest that targeting bacterial and viral determinants together could yield synergistic preventive or therapeutic benefits (Kyriakidis et al., 2025; Zeng et al., 2025; Rivera-Orellana et al., 2025; Bautista et al., 2025a).

At the translational level, interventions to shape the early-life microbiome are gaining momentum. Maternal strategies, including prebiotics, probiotics, synbiotics, and tailored dietary interventions during pregnancy and lactation, have shown promise in preclinical and early clinical trials (Reyman et al., 2019; Boudry et al., 2021; Tian M. et al., 2023). Postnatal approaches, such as vaginal microbial transfer, human milk oligosaccharide fortification, and fecal microbiota transplantation in neonatal intensive care units, are also being explored (Schwartz et al., 2020; Senn et al., 2020). However, robust clinical trials are needed to validate their safety, efficacy, and long-term impact.

Despite these advances, critical gaps persist. It remains unclear which microbial taxa or consortia confer resilience, how inter-individual variability shaped by genetics, maternal health, and geography modifies outcomes, or which biomarkers reliably predict disease risk (Senn et al., 2020; Wang et al., 2024). Incomplete functional annotation of microbial “dark matter” and lack of standardized protocols further hinder translation. Addressing these challenges requires longitudinal, multi-omic studies across diverse populations, integrating metagenomics, transcriptomics, metabolomics, and epigenomics to capture the complexity of host–microbe interactions (Chetty and Blekhman, 2024; Bautista et al., 2025c).

In conclusion, the perinatal microbiome represents a powerful determinant of human health with broad implications for preventive medicine. Future progress requires moving beyond descriptive associations toward translational frameworks that can guide clinical practice. Key priorities include: (1) the development of validated early-life microbial biomarkers for predicting risk of chronic disease; (2) the establishment of standardized definitions and metrics of neonatal dysbiosis to improve reproducibility across studies; (3) the creation of systems biology models that integrate host genetics, microbial ecology, and environmental exposures to capture the complexity of perinatal development; and (4) the harmonization of protocols for microbiome-based interventions, including probiotics, synbiotics, and FMT, to ensure safety and comparability across clinical trials (Reyman et al., 2019; Schwartz et al., 2023; Coenye et al., 2024; Porcari et al., 2025). By systematically addressing these gaps, the field will be better positioned to design precision microbiome-based strategies that reprogram developmental trajectories at the earliest stages of life and deliver lasting benefits across the lifespan.
